# Supplementary material for: Protein NirP1 regulates nitrite reductase and nitrite excretion in cyanobacteria
Source: Nat Commun. 2024 Mar 1;15:1911. doi: 10.1038/s41467-024-46253-4 (PMC10907346; doi:10.1038/s41467-024-46253-4)
Supplement: Supplementary file 1 — Supplementary Information File [file 41467_2024_46253_MOESM1_ESM.pdf]

Protein NirP1 regulates nitrite reductase and nitrite excretion in cyanobacteria

Alexander Kraus[1], Philipp Spät[2], Stefan Timm[3], Amy Wilson[1], Rhena Schumann[4], Martin Hagemann[3], Boris Macek[2], Wolfgang R. Hess[1]

[1]Genetics and Experimental Bioinformatics, Faculty of Biology, Freiburg University, D-79104 Freiburg, Germany;
[2]Department of Quantitative Proteomics, Interfaculty Institute for Cell Biology, University of Tübingen, D-72076 Tübingen, Germany;
[3]Plant Physiology Department, Institute of Biosciences, University of Rostock, D-18059 Rostock, Germany;
[4]Biological Station Zingst, University of Rostock, D-18374 Zingst, Germany

Correspondence to:

Wolfgang R. Hess: wolfgang.hess@biologie.uni-freiburg.de

Supplementary Information

Content:

Supplementary Figures: p. 2
Supplementary Tables: p. 12
Supplementary References: p. 15

Supplementary Figures

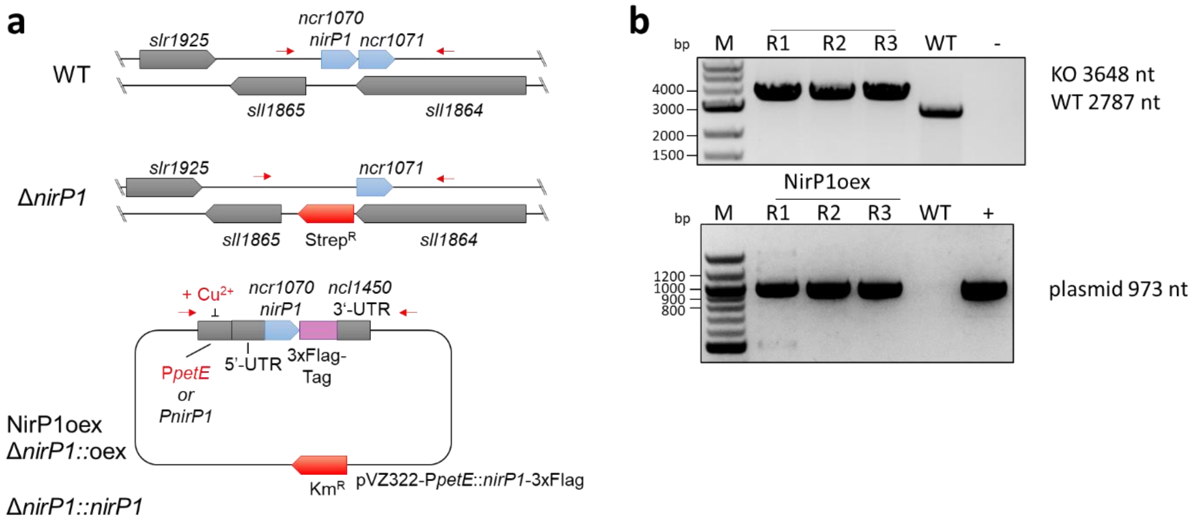

Fig. S1. Generation of Δ*nirP1* and *nirP1*-overexpressing strains. a The *nirP1* locus in the wild type and in the Δ*nirP1* deletion strain, as well as of a pVZ322 plasmid derivative harboring a *nirP1* gene copy under the control of the Cu^{2+}-inducible P_{petE} promoter in the *nirP1* overexpression strains (NirP1oex and Δ*nirP1::*oex) and the complementation strain (Δ*nirP1:: nirP1*) under the control of its native promoter. The *nirP1* gene was previously assigned to the transcriptional unit (TU) 2296 (1) extending from position 2215954 to 2217039 on the forward strand of the *Synechocystis* 6803 chromosome (GenBank accession no. NC_000911). The first segment of TU2296 has also been annotated as ncr1070 due to its sometimes-divergent accumulation compared to the second half, overlapping gene *sll1864* on the reverse strand. In the Δ*nirP1* strain, the gene was replaced by a streptomycin resistance cassette ($Strep^R$) via homologous recombination. The plasmid enabling ectopic *nirP1* expression was introduced into *Synechocystis* wild type and Δ*nirP1* strains. The red arrows indicate primer binding sites used to verify the mutants. **b** PCR verification of the genotypes of independently obtained mutant strains. Three clones were tested each, using primers P_AK9/P_AK10 (for Δ*nirP1*) or P_AK16/ P_AK17 (overexpression and complementation strains NirP1oex, Δ*nirP1*::oex and Δ*nirP1*::*nirP1*; primer sequences in **Table S1**). The PCR of NirP1oex representative for all pVZ322-containing strains is shown. Abbreviations: M, marker; bp, base pairs; c., clone; -, negative control (water control); +, positive control (purified plasmid as template). This figure is an extension to **Fig. 1** and illustrates technical aspects. Source data are provided as a Source Data file.

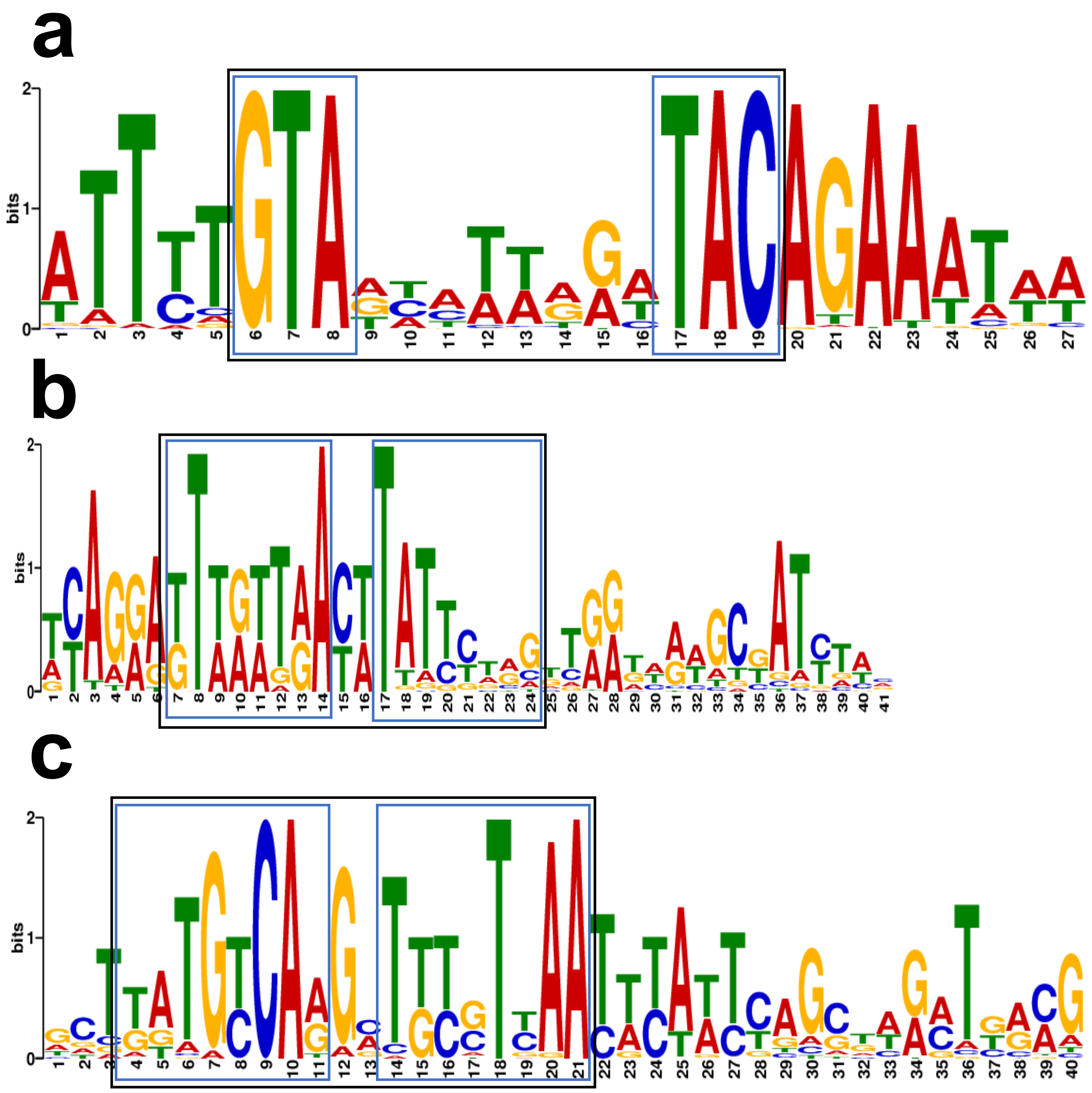

Fig. S2. Sequence similarities in the *nirP1* promotor region and promotor binding motifs. a The promotor region (sequences from -300 to +10, with +1 as the TSS) of 456 *nirP1* homologues from **Supplementary Dataset 1** were used for sequence alignment. MEME was used to illustrate the conserved motifs. The NtcA binding motif is shown in a black box, the important nucleotides for transcription factor binding in a blue box. Details of the motifs are given in **Supplementary Dataset 2**. **b** Sequence alignment of the tandem repeat. **c** Sequence logo of 135 homologs that have the tandem repeat motif in **Supplementary Dataset 2**.

These data represent an extension to the results shown in **Fig. 1** and **Fig. 2**.

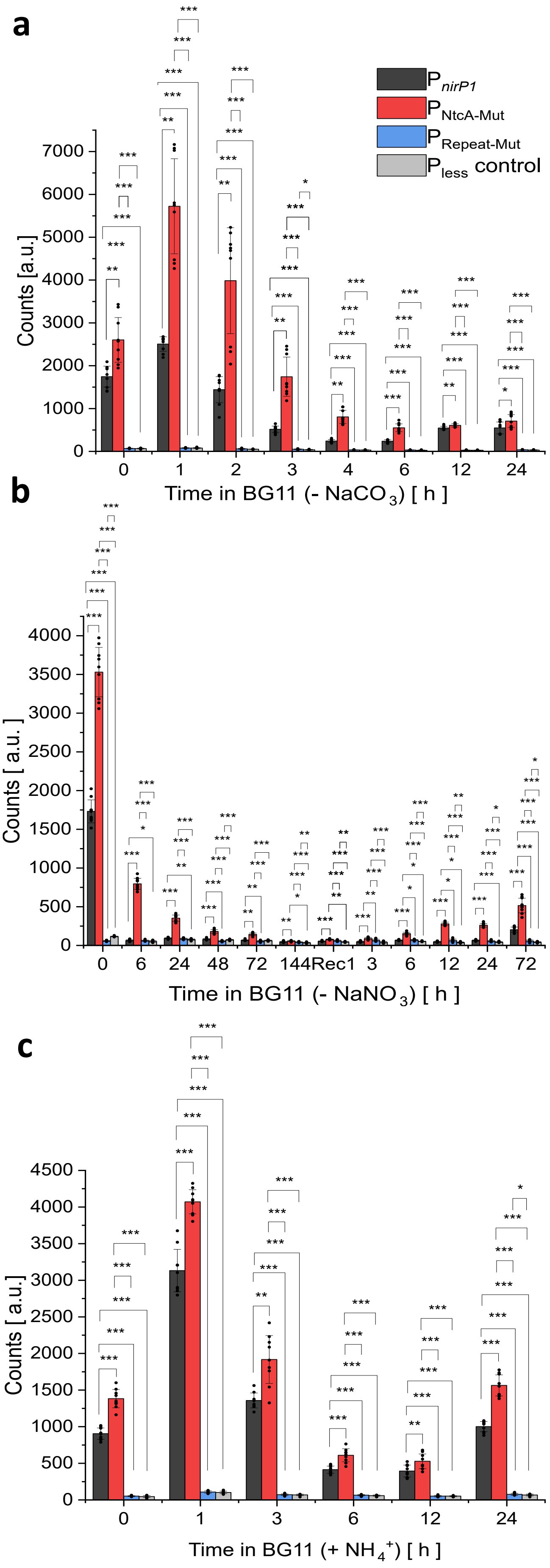
a
b
c
PnirP1
PNtcA-Mut
PRepeat-Mut
Pless control
Counts [a.u.]
Time in BG11 (- NaCO3) [h]
Counts [a.u.]
Time in BG11 (- NaNO3) [h]
Counts [a.u.]
Time in BG11 (+ NH4+) [h]

Fig. S3. NirP1 expression is mediated through NtcA and a C_i-sensitive promoter. **a** Bioluminescence of the *Synechocystis* 6803 reporter strains. Initially, cells were grown under HC for 2 h and transferred to BG11 medium without CO_2 source to induce LC conditions for 24 h. To improve the signal of bioluminescence mediated through the promoters, 2 µL decanal were added to the measurements. Bioluminescence data are presented as the means ± SD of n independent measurements for three biological replicates each (resulting from three independent transformants). In each experiment, a strain carrying a promoterless (pless) *luxAB* was used as a negative control (in each case measured in three independent cultures, n=3). **b** Cells were cultivated in standard conditions and subsequently transferred to NO_3-free BG11 medium to start nitrogen deprivation. After 7 days (time point 144 h) of nitrogen starvation, bleached cells were transferred back to standard BG11 medium (indicated as Rec) to start the recovery. **c** Cells were grown in standard conditions T_0 and were further grown with additional with 10 mM NH_4Cl for 24 h. Significance was calculated with a two-sample *t* test with unequal variance (Welch's *t* test; *, $P < 0.05$; **, $P < 0.01$; ***, $P < 0.001$) between the strains at corresponding time points. Further details of statistical analysis are given in **Supplementary Dataset 3**.

These data represent an extension to the results shown in **Fig. 2**.

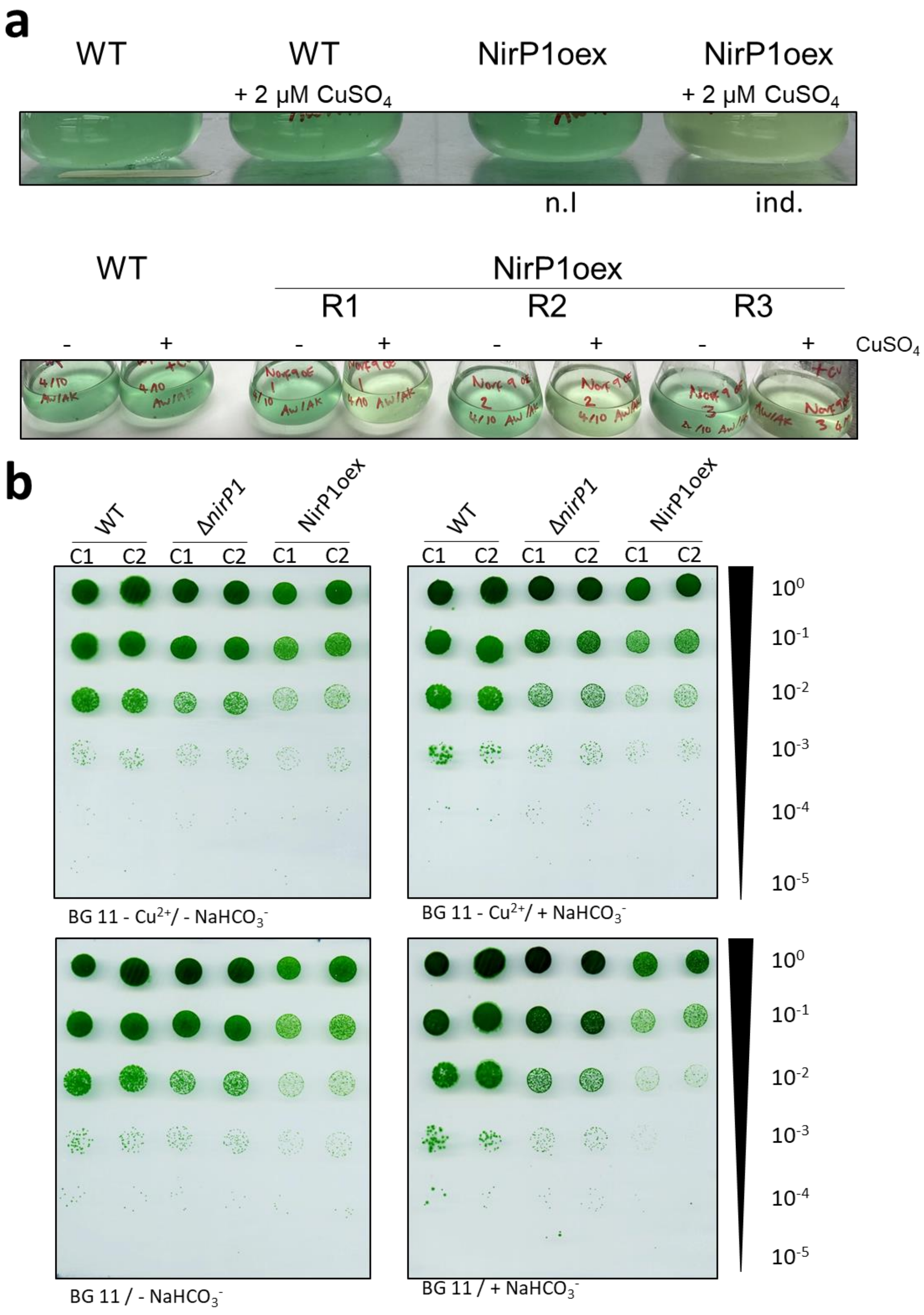

Fig. S4. Phenotypical differences between *nirP1* mutants and the wild type. a Pigmentation phenotype of wild type and *nirP1* overexpressor (NirP1oex) without and with 2 µM Cu_2SO_4 to trigger the expression of *nirP1* for 24 h from the P_{petE} promoter. Lower panel: Repetition with three biological replicates of NirP1oex (R1 to R3). WT was used as negative control. **b** Drop dilution assay on solid medium comparing

duplicate samples of the *Synechocystis* wild type (WT), the *nirP1* deletion mutant Δ*nirP1* and overexpression strain NirP1oex under continuous light in LC (without $NaHCO_3$) and HC (with 10 mM $NaHCO_3$) conditions. Strains were pre-cultivated in liquid BG11 medium without Cu_2SO_4 and without $NaHCO_3$ under constant light, and the indicated different dilutions were spotted on agar plates. For ectopic overexpression of NirP1, standard BG11 medium containing 0.3 µM Cu_2SO_4 was used in the plates. Plates were analyzed after incubation for 5 days. Source data are provided as a Source Data file.

These data represent an extension to the results shown in **Fig. 3**.

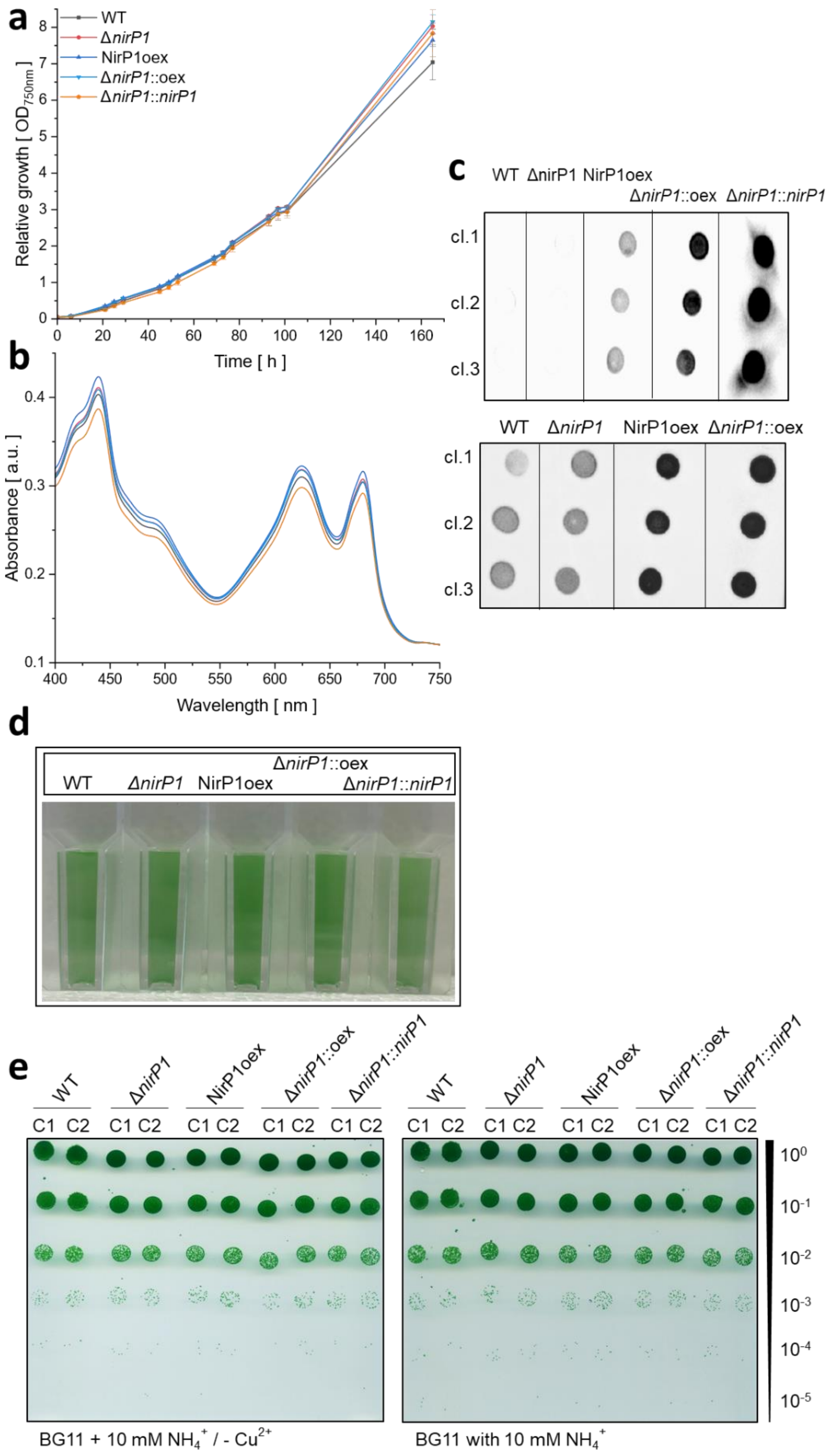

Fig. S5. Phenotypical differences with ammonium as the only nitrogen source. a Growth of *Synechocystis* wild type (black), the Δ*nirP1* deletion mutant (red), the strains expressing *nirP1* under control of the P_{petE} promoter in the wild type background (dark blue, NirP1oex) and Δ*nirP1* background (light blue, Δ*nirP1*::oex) and the

complementation strain (orange, Δ*nirP1*::*nirP1*) in medium with ammonia as the only nitrogen source. Data points represent the average of 3 biological replicates (n = 3). **b** Room temperature absorption spectra for wild-type and *nirP1* mutants expressing *nirP1* for 48 h from the native P_{nirP1} and P_{petE} promoter in the presence of Cu_2SO_4, with ammonium as the only nitrogen source normalized to wild-type OD_{750}. Same strains and colors as in panel a. **c** Western Dot blot of cell extract of *nirP1* mutant strains grown in medium with ammonium and Cu_2SO_4. Same samples as in panel a. Samples were taken after 7 days of cultivation. Lower panel: Same western dot blot without the signal of the complementation strain. **d** Pigmentation phenotype of wild type and *nirP1* overexpressor in the presence of Cu_2SO_4 expressing *nirP1* for 48 h. Same strains as in panel a. All cultures were set to an OD of ~0.8. **e** Drop dilution assay on solid medium comparing duplicate samples of the wild type (WT), the *nirP1* deletion mutant Δ*nirP1*, overexpression strains NirP1oex and Δ*nirP1*::oex and the complementation strain Δ*nirP1*::*nirP1* under continuous light with ammonia as nitrogen source. Strains were pre-cultivated in liquid BG11 medium without Cu_2SO_4 under constant light, were washed with medium lacking any nitrogen source and copper and the indicated different dilutions were spotted on agar plates containing 10 mM NH_4Cl. For ectopic overexpression of NirP1, medium containing 0.3 µM Cu_2SO_4 was used in the plates. Plates were analyzed after incubation for 5 days**.** Source data are provided as a Source Data file.

These data represent an extension to the results shown in **Fig. 3**.

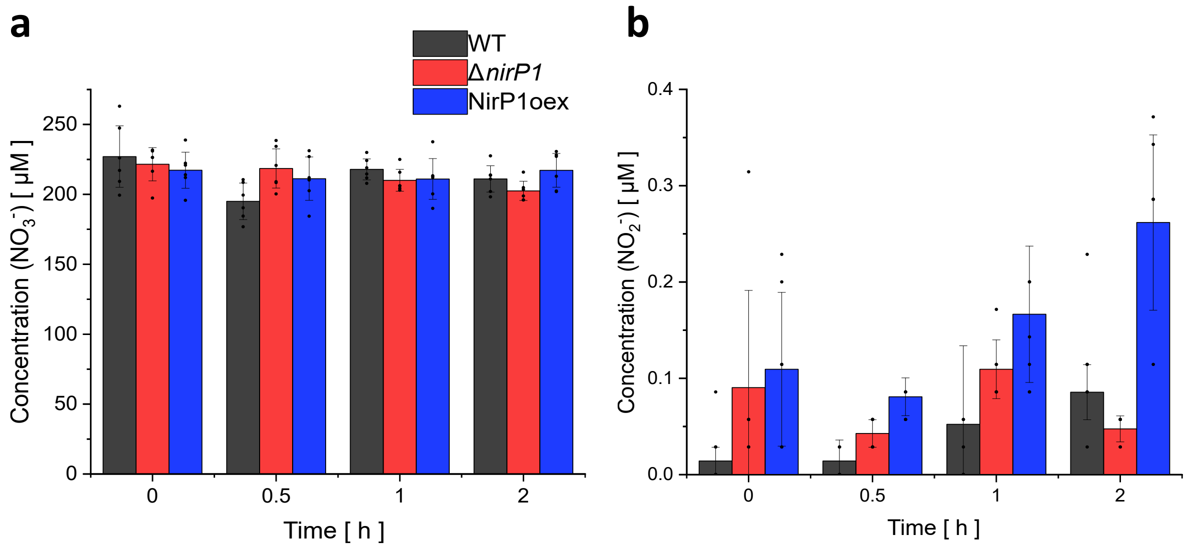

Fig. S6. Uptake of NO_3 and secretion of NO_2 in the presence of ammonia. **a** Uptake of NO_3. **b** Secretion of NO_2. The strains were grown in BG11, washed with nitrogen-free BG11 (BG11 –N), set to OD = 1 and resuspended in BG11 containing 200 µM nitrate and 2 mM ammonium. Concentration of nitrate and nitrite was measured in the supernatant after centrifugation of the cell suspensions. The data are presented as the means ± SD of 2 independent measurements for three biological replicates each (resulting from independent transformants, n = 6). Data details and statistical analysis of nitrate consumption and the nitrite excretion assay are given in **Supplementary Dataset 10**.

This is a control experiment to **Fig. 6**.

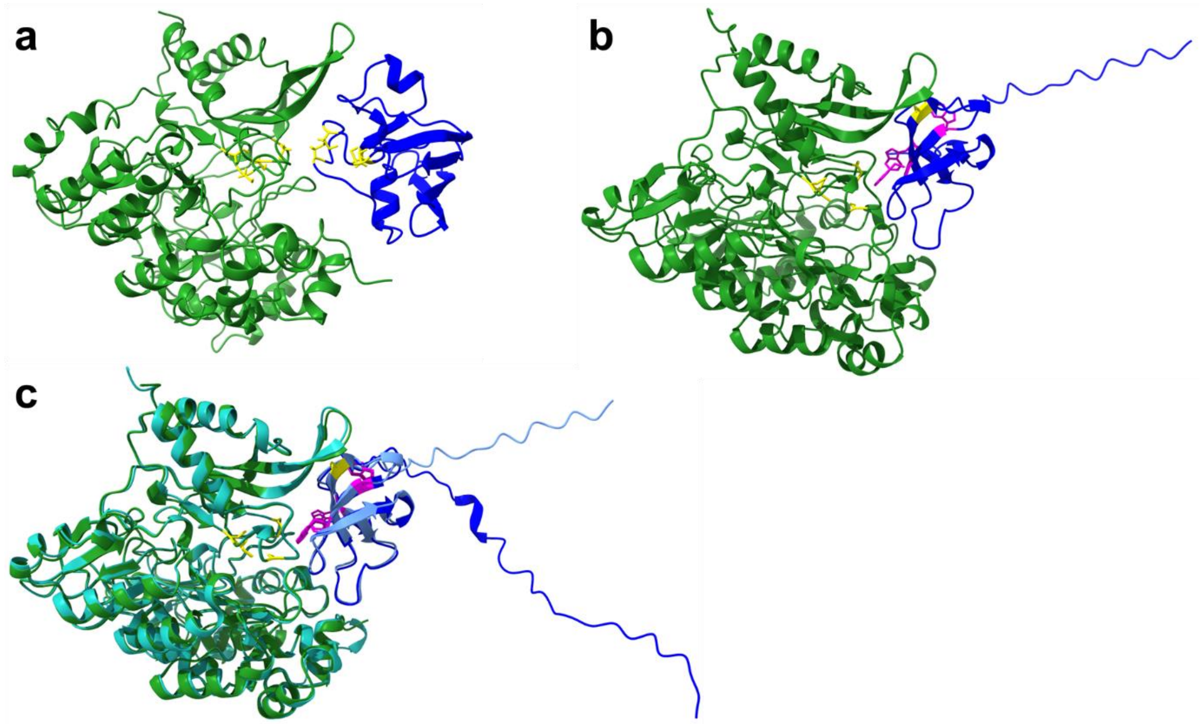

Fig. S7. Predicted NiR interactions with Fd1 in *Synechocystis* 6803 and with NirP1 in *Synechococcus* 7942. a Predicted interaction of the 97 AA sequence WP_190595418.1 for Fd1 (*ssl0020, petF*) and the 502 AA sequence WP_010873675.1 for NiR from *Synechocystis* 6803 using AlphaFold (2, 3). Both structures were modeled from the first to the last AA. The residues of the iron-sulfur clusters in each protein are highlighted in yellow. **b** Predicted interaction between the *Synechococcus elongatus* sp. PCC 7942 NiR-NirP1 homologs generated by AlphaFold using the 75 AA sequence WP_011244559.1 for NirP1 and the 512 AA sequence WP_011242624.1 for NiR. The residues of the iron-sulfur clusters in NiR are highlighted in yellow and the conserved AA in the NirP1 homologue as in **Fig. 5e** for the *Synechocystis* 6803 NirP1. **c** Overlay of the predicted interactions between NirP1 and NiR from *Synechocystis* 6803 *(*NiR in green and NirP1 in blue*)* and *Synechococcus (*NiR in black and NirP1 in orange*)* showing similar arrangements in the two different organisms.

This Figure is an extension to **Fig. 5e.**

Supplementary Tables

Table S1. Desoxyoligonucleotide primers used in this study (abbreviations: AQ, AQUA cloning (4)).

**Name**	**Sequence (5‘ to 3‘)**	**Description**	**Purpose**
P_AK1	CACAGCTTGTACTTAGCTTTGAG AATGG	AQ of *nirP1* KO construct to create flank1 (F1) with overlap to puC19 plasmid backbone	Generation of the *nirP1* knock out strain with pUC19 plasmid
P_AK2	TACGATAATATTACAAAATGATA GGGATAATAATTTG	AQ of *nirP1* KO to create flank1 with overlap to the streptomycin resistance cassette ($Strep^R$)	
P_AK3	CATTTTGTAATATTATCGTAGTT GCTCTCAG	AQ of *nirP1* KO to create the $Strep^R$ cassette with overlap to flank 1	
P_AK4	CTTGGGCAGCGTACAGAGTGAT GTCAAC	AQ of *nirP1* KO to create the $Strep^R$ cassette with overlap to flank 2	
P_AK5	CACTCTGTACGCTGCCCAAGCT AGAATC	AQ of *nirP1* KO to create flank 2 (F2) with overlap to the $Strep^R$ cassette	
P_AK6	CCGCTTACAGCCCCCAATCCCA TCACTG	AQ of *nirP1* KO to create flank 2 with overlap to the $Strep^R$ cassette	
P_AK7	GGATTGGGGGCTGTAAGCGGAT GCCGGG	AQ of *nirP1* KO to create the backbone of puC19 with overlay to flank 2	
P_AK8	AAAGCTAAGTACAAGCTGTGAC CGTCTC	AQ of *nirP1* KO to create the backbone of puC19 with overlay to flank1	
P_AK9	CATGTCCCCTCGAATTTC	Segregation check for *nirP1* deletion	
P_AK10	GTGCACATTGCCCTTAAG	Segregation check for *nirP1* deletion	
P_AK11	GACATTAACCTATAAAAATAGGC G	Sequencing the flank 1 of *nirP1* KO construct in puc19	
P_AK12	GGCAGGTCAAAGATAGTC	Sequencing the $Strep^R$ cassette of *nirP1* KO construct in puc19	
P_AK13	GATCAAAGAGTTCCTCCG	Sequencing the $Strep^R$ cassette of *nirP1* KO construct in puc19	
P_AK14	GCGGAAAATAAACACAGTGG	Sequencing flank 2 of *nirP1* KO construct in puc19	
P_AK15	GTAATAAAATGCGCCCTTC	Sequencing the last part of flank 2 of *nirP1* KO construct in puc19	
P_AK16	CTGCCCGGATTACAGATC	Sequencing the *nirP1* constructs on the pVZ322 plasmid	Construction of pVZ322::$P_{nirP1/petE}$::nirP1::3xFLAG for ectopic expression in *Synechocystis*
P_AK17	GTAATACCATGAAAAATACCATG CTCAG	Sequencing the *nirP1* constructs on the pVZ322 plasmid	
P_AK18	GGATTACAGATCCTCTAGAGACT TATCGTGTTTGTCAAG	Fragment for the native promotor of *nirP1* with the overlap to the pVZ322 plasmid	

P_AK19	AGGTTCATTTGTGGCGACATAAAGTCCTCTCCATTTTAC	Fragment for the native promotor of *nirP1* with the overlap to the pVZ322 plasmid	
P_AK20	ATGTCGCCACAAATGAACC	Primer for the NirP1 ORF at the translational start site	
P_AK21	CCATCATGATCTTTATAATCACAATAAAAATCACTGCGGTC	Primer for the NirP1 ORF	
P_AK22	TTACAGATCCTCTAGAGTCGACCTGGGCCTACTGGGCTATTC	Fragment for the *petE* promotor of *nirP1* with the overlap to the pVZ322 plasmid	
P_AK23	GGTTCATTTGTGGCGACATACTTCTTGGCGATTGTATC	Fragment for the petE promotor of *nirP1* with the overlap to the pVZ322 plasmid	
P_AK24	GATTATAAAGATCATGATGG	3xFlagTag and 3'UTR for taggig small proteins	
P_AK25	GATGTATGCTCTTCTGCTCCTGCAGTAATAAAAAACGCCCGGCGGCAACCGAGCGAATAATTCCCAACGAAGGCAAGC	3xFlagTag and 3'UTR for taggig small proteins	
P_AK26	GTGCAGGTCGACTCTACCGGACTTATCGTGTTTGTCAAGATTTG	Promotor site of *nirP1* (region from -70 to + 30)	pILA vectors with *nirP1* promotor site and variants for biolumi-nescence assays
P_AK27	TCATCTAATGCTAAGGCCGGAAGTCAAAGGAATAATTTCTGTATCC	Promotor site of *nirP1* (region from -70 to + 30)	
P_AK28	TCATCTAATGCTAAGGCCGGAAGTCAAAGGAATAATTTCTGATTCCATATTTAGAAAATG	Promotor site of *nirP1* with mutation in the NtcA binding site (region from -70 to + 30)	
P_AK29	GTGCAGGTCGACTCTACCGGACTTATCGTGTTTGTCCCGAGGGCTTAATTTTG	Promotor site of *nirP1* with mutation in the tandem repeat (region from -70 to + 30)	
P_AK30	TAATACGACTCACTATAGGCTTTAGCACTTATCCGGG	Probe for *nirP1* northern hybridization with T7 promoter	Probe for northern hybridization
P_AK31	ATATGGATACAGAAATTATTCC	Probe for *nirP1* northern hybridization	

Table S2. Plasmids used in this study.

**Plasmid name**	**Description**	**Reference**
pUC19	High-copy cloning vector used for replication in *E. coli*, Amp^R	(5)
pUC19::*nirP1*_KO	Plasmid used for homologous recombination of kan^R cassette into the *nirP1* locus using up- and downstream genomic regions flank 1 and flank 2 as flanking sites to create the KO strain Δ*nirP1*	This study
pVZ322::P_{nirP1}::*nirP1*::3×FLAG	Self-replicating, conjugative plasmid for native *nirP1* expression in *Synechocystis* 6803 driven by P_{nirP1} promoter, Km^R & Gen^R	This study
pVZ322::P_{petE}::*nirP1*::3×FLAG	Self-replicating, conjugative plasmid for copper inducible *nirP1* expression from P_{petE} promotor in *Synechocystis* 6803, Km^R & Gen^R used to create the expression strains NirP1oex and Δ*nirP1*::*nirP1*	This study
pilA (FseI, AgeI)	Plasmid used for homologous recombination of promotor regions fused to *luxAB* genes; used for bioluminescence assay	(6, 7)
pILA::P_{nirP1}::*luxAB*	Plasmid to fuse the *nirP1* promotor region to *luxAB* genes for bioluminescence assay	This study
pILA::$P_{NtcA\text{-}Mut}$::luxAB	Plasmid to fuse the *nirP1* promotor region with mutation in the NtcA binding site to *luxAB* for bioluminescence assay	This study
pILA::$P_{Repeat\text{-}Mut}$::luxAB	Plasmid to fuse the *nirP1* promotor region with mutations in the upstream binding motif to *luxAB* for bioluminescence assay	This study

Supplementary References

1. M. Kopf, *et al.*, Comparative analysis of the primary transcriptome of *Synechocystis* sp. PCC 6803. *DNA Res.* **21**, 527–539 (2014).

2. J. Jumper, *et al.*, Highly accurate protein structure prediction with AlphaFold. *Nature* **596**, 583–589 (2021).

3. M. Mirdita, *et al.*, ColabFold: making protein folding accessible to all. *Nat. Methods* **19**, 679–682 (2022).

4. H. M. Beyer, *et al.*, AQUA cloning: a versatile and simple enzyme-free cloning approach. *PLOS ONE* **10**, e0137652 (2015).

5. C. Yanisch-Perron, J. Vieira, J. Messing, Improved M13 phage cloning vectors and host strains: nucleotide sequences of the M13mp18 and pUC19 vectors. *Gene* **33**, 103–119 (1985).

6. A. Kunert, M. Hagemann, N. Erdmann, Construction of promoter probe vectors for *Synechocystis* sp. PCC 6803 using the light-emitting reporter systems Gfp and LuxAB. *J. Microbiol.l Meth.* **41**, 185–194 (2000).

7. S. Klähn, *et al.*, Alkane biosynthesis genes in cyanobacteria and their transcriptional organization. *Front. Bioeng. Biotechnol.* **2**, 24 (2014).
