# Supplementary material for: Protein NirP1 regulates nitrite reductase and nitrite excretion in cyanobacteria
Source: Nat Commun. 2024 Mar 1;15:1911. doi: 10.1038/s41467-024-46253-4 (PMC10907346; doi:10.1038/s41467-024-46253-4)
Supplement: Supplementary file 3 — Description of Additional Supplementary Files [file 41467_2024_46253_MOESM3_ESM.pdf]

Description of Additional Supplementary Files:

Supplementary Dataset 1: Results of Blast searches for homologs of NirP1. Sequences of 485 detected homologs of NirP1.

Supplementary Dataset 2: Data for promotor sequence alignments.

Supplementary Dataset 3: Measurements and statistical analysis of the promoter bioluminescence assay.

Supplementary Dataset 4: Measurements of growth curves and absorption spectra under different conditions. Calculation of the growth rate and doubling time.

Supplementary Dataset 5: Measurements and statistical analysis of extracted metabolites.

Supplementary Dataset 6: Repetition of measurements and statistical analysis of metabolic changes due to NirP1 expression.

Supplementary Dataset 7: List of identified proteins from NirP1-3xFLAG Co-IP and corresponding LFQ intensities.

Supplementary Dataset 8: List of identified peptides of NirP1-3xFlag (Norf9-3xFlag).

Supplementary Dataset 9: List of identified peptides of NiR.

Supplementary Dataset 10: Measurement and statistical analysis of nitrate consumption and nitrite excretion assay.
