# Supplementary material for: Protein NirP1 regulates nitrite reductase and nitrite excretion in cyanobacteria
Source: Nat Commun. 2024 Mar 1;15:1911. doi: 10.1038/s41467-024-46253-4 (PMC10907346; doi:10.1038/s41467-024-46253-4)
Supplement: Supplementary file 9 — Reporting Summary [file 41467_2024_46253_MOESM9_ESM.pdf]

nature portfolio

Corresponding author(s): Wolfgang R. Hess

Last updated by author(s): Feb 16, 2024

Reporting Summary

Nature Portfolio wishes to improve the reproducibility of the work that we publish. This form provides structure for consistency and transparency in reporting. For further information on Nature Portfolio policies, see our Editorial Policies and the Editorial Policy Checklist.

Statistics

For all statistical analyses, confirm that the following items are present in the figure legend, table legend, main text, or Methods section.

n/a	Confirmed	
☐	☒	The exact sample size (*n*) for each experimental group/condition, given as a discrete number and unit of measurement
☐	☒	A statement on whether measurements were taken from distinct samples or whether the same sample was measured repeatedly
☐	☒	The statistical test(s) used AND whether they are one- or two-sided<br>*Only common tests should be described solely by name; describe more complex techniques in the Methods section.*
☐	☒	A description of all covariates tested
☐	☒	A description of any assumptions or corrections, such as tests of normality and adjustment for multiple comparisons
☐	☒	A full description of the statistical parameters including central tendency (e.g. means) or other basic estimates (e.g. regression coefficient) AND variation (e.g. standard deviation) or associated estimates of uncertainty (e.g. confidence intervals)
☐	☒	For null hypothesis testing, the test statistic (e.g. *F*, *t*, *r*) with confidence intervals, effect sizes, degrees of freedom and *P* value noted<br>*Give P values as exact values whenever suitable.*
☒	☐	For Bayesian analysis, information on the choice of priors and Markov chain Monte Carlo settings
☒	☐	For hierarchical and complex designs, identification of the appropriate level for tests and full reporting of outcomes
☒	☐	Estimates of effect sizes (e.g. Cohen's *d*, Pearson's *r*), indicating how they were calculated

Our web collection on statistics for biologists contains articles on many of the points above.

Software and code

Policy information about availability of computer code

Data collection	Mass spectrometry data were collected on an Orbitrap Exploris 480 mass spectrometer (Thermo Fisher Scientific, USA).<br>Absolute metabolite contents were quantified using the ultra-high performance liquid chromatograph mass spectrometer UHPLC-MS/MS-8050 system (Shimadzu) and the incorporated LC-MS/MS method package for primary metabolites (version 2, Shimadzu).<br>Raw absorption spectra were measured using a Specord® 250 Plus (Analytik Jena) and the ASpec UV software (version 1.2.1).<br>Bioluminescence was measured using 1420 Multilabel Counter VICTOR3 (Perkin Elmer) and the according Wallac 1420 Manager software (version 3.00).<br>Northern blot hybridisation was quantified with the Typhoon FLA 9500 (GE Healthcare Life Sciences) and the according Typhoon FLA 9500 software (version 1.1).<br>Western blot signals were detected with Fusion SL4 and the according Fusion Capt Advanced SL7 software (version17.04a).

Data analysis	Raw spectra during proteomics were processed with MaxQuant software (version 1.6.8.0) and further analyzed using the Perseus software suite (version 1.6.5.0).<br>Raw metabolite spectra were processed and interpreted using the Lab solution software package (Shimadzu).<br>Ion chromatography were quantified using the Chromeleon® 7 software.<br>To illustrate the promoter sequence alignments the web tool MEME (version 5.5.5) was used.<br>Northern blot and western blot signal intensities were calculated and analyzed with Quantity One (version 4.6.6).<br>All statistics were calculated with Excel, data and plots were visualized with OriginPro (2022) software (version 9.9.0.225) and vector images were created with Inkscape (version 1.2.1).<br>Protein sequences were aligned with tool Clustl Omega and alignments were illustrated using Jalview (version 2.11.3.2).<br>Protein structures were predicted using the AlphaFold web tool (version 2).

For manuscripts utilizing custom algorithms or software that are central to the research but not yet described in published literature, software must be made available to editors and reviewers. We strongly encourage code deposition in a community repository (e.g. GitHub). See the Nature Portfolio guidelines for submitting code & software for further information.

Data

Policy information about availability of data

All manuscripts must include a data availability statement. This statement should provide the following information, where applicable:

- Accession codes, unique identifiers, or web links for publicly available datasets
- A description of any restrictions on data availability
- For clinical datasets or third party data, please ensure that the statement adheres to our policy

The mass spectrometry proteomics data generated in this study have been deposited at the ProteomeXchange Consortium via the PRIDE59 partner repository with the dataset identifier PXD041127. The mass spectrometry data are available in the PRIDE database under accession code PXD041127 [https://www.ebi.ac.uk/pride/archive/projects/PXD041127]. Source data are provided with this paper.

Research involving human participants, their data, or biological material

Policy information about studies with human participants or human data. See also policy information about sex, gender (identity/presentation), and sexual orientation and race, ethnicity and racism.

Reporting on sex and gender	No research involving human research participants.
Reporting on race, ethnicity, or other socially relevant groupings	N/A
Population characteristics	N/A
Recruitment	N/A
Ethics oversight	N/A

Note that full information on the approval of the study protocol must also be provided in the manuscript.

Field-specific reporting

Please select the one below that is the best fit for your research. If you are not sure, read the appropriate sections before making your selection.

[x] Life sciences [ ] Behavioural & social sciences [ ] Ecological, evolutionary & environmental sciences

For a reference copy of the document with all sections, see nature.com/documents/nr-reporting-summary-flat.pdf

Life sciences study design

All studies must disclose on these points even when the disclosure is negative.

Sample size	The current work doesn't involve the analysis of populations. For functional assays, the number of times that experiments were repeated is addressed under "Replication" below.
Data exclusions	No data was excluded.
Replication	All biochemical and in vitro assays were repeated several times independently with at least duplicate samples per assay. The detailed replicates for each experiments are given in the manuscript. All attempts at replication were successful.
Randomization	Randomization was not relevant because our study did not involve the allocation of samples/organisms/participants into experimental groups.
Blinding	Investigators were not blinded to group allocation because group allocation was not involved in our study. Investigators were not blinded during data collection because the collected data were quantitative in nature (such as bands in gel blot analyses or fluorescence values) and were not prone to subjective interpretation.

Reporting for specific materials, systems and methods

We require information from authors about some types of materials, experimental systems and methods used in many studies. Here, indicate whether each material, system or method listed is relevant to your study. If you are not sure if a list item applies to your research, read the appropriate section before selecting a response.

Materials & experimental systems

n/a	Involved in the study
☐	☒ Antibodies
☒	☐ Eukaryotic cell lines
☒	☐ Palaeontology and archaeology
☒	☐ Animals and other organisms
☒	☐ Clinical data
☒	☐ Dual use research of concern
☒	☐ Plants

Methods

n/a	Involved in the study
☒	☐ ChIP-seq
☒	☐ Flow cytometry
☒	☐ MRI-based neuroimaging

Antibodies

Antibodies used	Anti-DYKDDDDK (FLAG tag) Monoclonal Antibody (Sigma, cat #SAB4301135) produced in rabbit at a dilution of 1:5,000.
Validation	Anti-DYKDDDDK (FLAG tag) Rabbit Monoclonal Antibody (#SAB4301135, 1:5,000, Sigma) has been used successfully used in a peer-reviewed paper (https://www.sigmaaldrich.com/DE/de/product/sigma/sab4301135).

Plants

Seed stocks	This study did not involve research on plants.
Novel plant genotypes	N/A
Authentication	N/A
